# Driving CAR-T cells toward solid lung tumors

**DOI:** 10.1016/j.omton.2024.200764

**Published:** 2024-02-07

**Authors:** Frank J.T. Staal, Federico Avila-Moreno

**Affiliations:** 1 Laboratory for Stem Cell Biology and Lymphocyte Development, Department of Immunology, L3-35, Department of Pediatrics, Leiden University Medical School (LUMC), Leiden, the Netherlands; 2 Research Unit, National Institute of Cancerology (INCAN), Mexico City, Mexico

## Main text

CAR-T (chimeric antigen receptor T) cell therapy has shown promising results in treating certain non-solid malignant tumors such as leukemia and B cell lymphoma.^1^ However, CAR-T cell therapy for solid tumors has been more challenging in part due to the immunosuppressive tumor microenvironment and the lack of specific antigens on the surface of solid tumor malignant cells.^2^ The presence of less differentiated T cell subpopulations within the prepared CAR-T products is associated with better clinical outcome response. Indeed, naive and memory T cells persist longer and exhibit greater antitumor activity than effector T cells. Especially, attention in recent years has gone to stem cell-like memory T (TSCM) cells in the T cell therapy field.^3^ This T cell subset has been reported as the least differentiated of the memory T cell subpopulations. TSCM cells have been recently identified as CD4+ or CD8+ T cells with a semi-naïve-like phenotype expressing CD45RA, CCR7, and higher levels of the transcriptional regulator T cell factor 1 (TCF-1).

In the December issue of Molecular Therapy Oncolytics (MTO),^4^ a dedicated team from GSK-Stevenage’s Cell and Gene Therapy division, under the leadership of Claudia Montiel Equihua, presented a comprehensive exploration into the phenotypic composition of the initial T cells and the resultant CAR-T cell products employed in the treatment of non-small cell lung cancer (NSCLC) patients.

This researcher’s team has explored a transcendental concern in the field of lung oncology termed systemic anti-cancer treatment (SACT), which classifies individual or combined chemotherapy and/or immunotherapy treatments applied on NSCLC patients. This classification has been originated in the clinic by the UK National Institute for Health and Care Excellence,^5^^,^^6^ taking into account the major EGFR and ALK mutational status, as well as the PD-1 gene expression level in NSCLC patients, and it has been evaluated by other oncological-medical systems worldwide.

Based on this scheme, the present work has described that the quality and therefore clinical success of adoptive T cell-based therapies is highly dependent on the initial T cell fitness and cellular subsets composition, which will likely differ with age, diseases, and comorbidities, as well as medical treatments with prior therapies. Here the authors were particularly concerned with the effects of several prior SACTs, identifying three patient subgroups: (1) patients treated with check-point blockers (mostly PD1/PD1L), (2) cisplatin-like chemotherapeutic agents, and (3) tyrosine kinase inhibitors. Although some differences in composition, especially in TSCM, were found, there were surprisingly few differences in the final CAR-T cell products made, with which the authors successfully induced antitumor immune response for n = 40 NSCLC patients.

Such observations implied that, at least *in vitro*, T cell products derived from cancer patients who underwent SACT exhibited similar behavior compared with those from healthy donor controls. However, two crucial considerations must be highlighted according to the authors: first, CAR-T cell products could not always be generated from subjects treated with an anti-PDL1 scheme, suggesting a deficiency in the proliferative capacity of T cells derived from these NSCLC patients. Second, although the CAR-T cell products appear comparable by *in vitro* analyses, their effects in the *in vivo* setting analysis when NSCLC patients were treated by PDL1-SACT-based therapy CAR-T cell products could be negatively affected. Nonetheless, this thorough investigation holds the potential to provide a novel variable to improve the clinical algorithms used in the establishment of SACT decisions reinforcing efficient therapy protocols for enhancing CAR-T cell products on NSCLC patients and/or other patients with diagnosis of solid malignant tumors.

Additionally, the present report provides essential quality control parameters crucial for predicting clinical success, mitigating negative impacts on both T cell phenotype, and most crucially, the anti-tumoral efficacy of adoptive t cell therapy (ACT). Based on this recent bioengineered therapy, which is crafted or derived from autologous T cells sourced from patients with lung malignant disease, whose knowledge has recently been employed as a cutting-edge technology applied as cellular and genetic therapy, it has facilitated an improved transduced T cell product through the CAR-T transduced methods by use of lentiviral vectors.

So, how could CAR-T developers improve their therapies based on these insights? One obvious approach is further purification of relevant T cell subsets, such as TSCM, but this may not be so easily translated to a clinical setting, as methods to purify rare subsets are difficult to scale up under good manufacturing practice conditions, and the actual cell numbers needed for clinical transplantation are largely unknown. A possible alternative strategy comes from epigenetic modulation of the CAR-T cell product. Recently, it has been highlighted how failures in the ACT strategies in solid malignant tumors might be overcome through inactivation of the H3K9 tri-methyltransferase. Inactivation of the H3K9 tri-methyltransferase SUV39H1 enhances CAR-T cell stem/memory differentiation and leads to long-term persistence, protecting from the tumor relapses and rechallenges in lung and disseminated solid tumor models.^7^ Based on the insight that epigenetically reprogrammed CAR-T cells are promoting a better self-renewing of potential stem-like populations, this may be a promising, integrative approach for driving CAR-T cell therapy toward solid human lung tumors.
